# Impact of accelerometer data processing decisions on the sample size, wear time and physical activity level of a large cohort study

**DOI:** 10.1186/1471-2458-14-1210

**Published:** 2014-11-24

**Authors:** Sarah Kozey Keadle, Eric J Shiroma, Patty S Freedson, I-Min Lee

**Affiliations:** Nutritional Epidemiology Branch, Division of Cancer Epidemiology and Genetics, National Cancer Institute, Bethesda, MD USA; Cancer Prevention Fellowship Program, Division of Cancer Prevention, National Cancer Institute, Bethesda, MD USA; Department of Epidemiology, Harvard School of Public Health, Boston, MA USA; Division of Preventive Medicine, Brigham and Women’s Hospital, Harvard Medical School, Boston, MA USA; Department of Kinesiology, School of Public Health and Health Sciences, University of Massachusetts Amherst, Amherst, MA USA

**Keywords:** Physical activity, Measurement, Exposure assessment, Behavioral epidemiology, Sedentary behavior

## Abstract

**Background:**

Accelerometers objectively assess physical activity (PA) and are currently used in several large-scale epidemiological studies, but there is no consensus for processing the data. This study compared the impact of wear-time assessment methods and using either vertical (V)-axis or vector magnitude (VM) cut-points on accelerometer output.

**Methods:**

Participants (7,650 women, mean age 71.4 y) were mailed an accelerometer (ActiGraph GT3X+), instructed to wear it for 7 days, record dates and times the monitor was worn on a log, and return the monitor and log via mail. Data were processed using three wear-time methods (logs, Troiano or Choi algorithms) and V-axis or VM cut-points.

**Results:**

Using algorithms alone resulted in "mail-days" incorrectly identified as "wear-days" (27-79% of subjects had >7-days of valid data). Using only dates from the log and the Choi algorithm yielded: 1) larger samples with valid data than using log dates and times, 2) similar wear-times as using log dates and times, 3) more wear-time (V, 48.1 min more; VM, 29.5 min more) than only log dates and Troiano algorithm. Wear-time algorithm impacted sedentary time (~30-60 min lower for Troiano vs. Choi) but not moderate-to-vigorous (MV) PA time. Using V-axis cut-points yielded ~60 min more sedentary time and ~10 min less MVPA time than using VM cut-points.

**Conclusions:**

Combining log-dates and the Choi algorithm was optimal, minimizing missing data and researcher burden. Estimates of time in physical activity and sedentary behavior are not directly comparable between V-axis and VM cut-points. These findings will inform consensus development for accelerometer data processing in ongoing epidemiologic studies.

**Electronic supplementary material:**

The online version of this article (doi:10.1186/1471-2458-14-1210) contains supplementary material, which is available to authorized users.

## Background

Physical activity (PA) is important for maintaining physical function, reducing morbidity due to chronic diseases and increasing longevity [1]. The majority of evidence is from studies using self-report questionnaires, which have numerous advantages including low participant and researcher burden and low cost [2, 3]. However, questionnaires are imprecise [4], in particular for assessing low-intensity activities, which are the predominant form of PA for older adults [5]. Activity monitors, including accelerometers, are an alternative or complementary method to assess PA [3] and decreases in cost of these monitors has increased feasibility for their use in epidemiologic studies [6]. However, in a recent review, Lee and Shiroma outlined logistic, data processing, and analysis challenges encountered when implementing accelerometers in a large-scale study [7]. Data processing decisions may impact the sample size available for analysis and summary estimates (e.g., moderate-to-vigorous (MV) PA) [8, 9]. To our knowledge this impact has not been quantified among adults. In the current study, two fundamental data processing decisions that influence estimates of PA and sedentary behavior using the commercially available ActiGraph GT3X+ (ActiGraph, Inc, Pensacola, FL) accelerometer were investigated.

The first decision relates to determining the time that the accelerometer was worn, commonly called "wear-time". Traditionally, logs are used where the participant records the dates and times the monitor was put on and taken off [6]. Even in small samples, it is burdensome to code and process these data, and use of logs may not be superior to automated procedures, which apply a computer algorithm to the data to estimate wear-time [5, 10, 11]. Many epidemiologic studies employ a mail-based protocol (for feasibility and cost reasons) where participants are sent study materials and return them in the mail. This mail-based protocol has been implemented with activity monitors in a few epidemiological cohorts [7], though to date it is not known how this protocol influences wear-time estimated using automated algorithms.

The second decision relates to differences in output when data from the vertical axis (counts per minute, cpm) only are used, compared to using data collected from 3 axes (vertical, anterior-posterior and medio-lateral) and combined into a vector magnitude (VM) score (square root of the sum of squares of cpm from all 3 axes). Many studies rely on the vertical axis cpm to estimate PA and sedentary behavior, although the additional information included in VM output may enhance precision [5, 12]. To our knowledge, summary estimates (e.g., wear-time; sedentary and MVPA time) using the vertical axis alone compared to the VM have not been explored in free-living samples.

Currently, several epidemiologic studies use accelerometers to measure PA in several thousand participants [7, 13]. The lack of consensus on data processing is inefficient and will limit the ability to compare data across studies. This paper therefore aims to provide empirical evidence in a large sample to inform and help advance consensus development on standard, best practices for data processing.

## Methods

### Study participants

Participants were from the Women’s Health Study (WHS), a completed randomized trial (1992 – 2004) of aspirin and vitamin E for preventing cardiovascular disease and cancer among 39,876 healthy women aged >45 years [14–16]. When the trial ended, 33,681 women (89% of those alive) consented to continue with observational follow-up, reporting on their health habits and medical history annually on questionnaires. In 2011, data collection began for an ancillary study, whose main aim was to examine accelerometer-measured PA and sedentary behavior in relation to health outcomes. Women provided written consent to participate and the study was approved by the Brigham and Women’s Hospital’s institutional review board committee.

**Figure 1:**
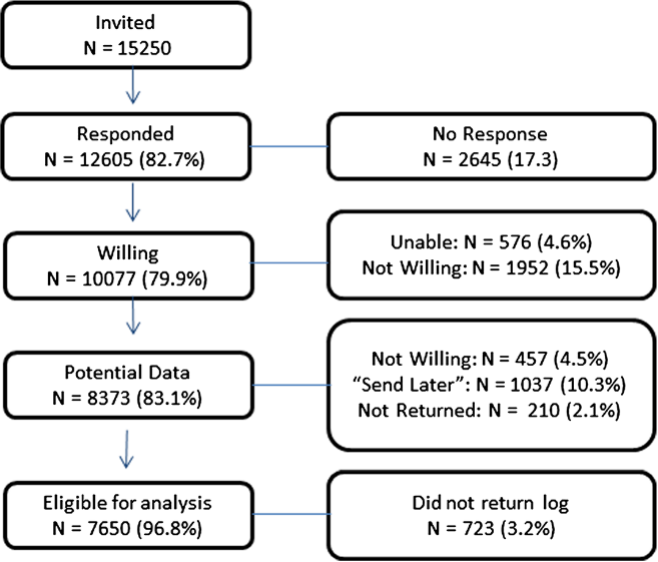
**Flow-chart of participants invited to participate in study.**

The present study includes 8,373 women who returned their accelerometers by March 2013 (approximately half of the estimated total sample). 723 women who did not return a log were excluded, leaving 7,650 eligible. Figure 1 illustrates the flow of participants invited to participate through to the eligible sample. Women were mailed an accelerometer ActiGraph GT3X+ (ActiGraph, Inc, Pensacola, FL) and asked to wear the monitor, secured with an adjustable belt on the hip, for 7 consecutive days during waking hours. The monitors were initialized to begin collecting data one day before the United States Postal Service estimated delivery date and to continue recording data until they were downloaded. Participants were provided a paper log to record the dates and times that the accelerometer was put on and taken off (See Additional file 1, Monitor-log). After the 7 days of wear, women were asked to return the monitor and log by mail using a prepaid return envelope. Raw data collected by the accelerometer were integrated into 60-second epochs using ActiLife software with the normal filter option [17] and expressed as cpm.

### Wear-time estimation

Wear-time was determined using three different methods. The first used monitor-logs, where participants recorded the date, time they woke up in the morning, time monitor was put on, time monitor was taken off, and time to bed at night. This composite set of data is referred to as the "detailed-log" data.

The second method used the algorithm described by Troiano et al. for processing data collected in NHANES [5]. For vertical axis data, non-wear time is defined as 60 consecutive minutes of 0 cpm, with allowance for 1-2 minutes of 0-99 cpm during this time. This algorithm is provided in the ActiLife software (from the Actigraph manufacturer) and also is available at http://riskfactor.cancer.gov/tools/nhanes_pam/. For VM data, the algorithm was modified to allow for 1-2 minutes of counts between 0-200 cpm within the 60 consecutive minutes of 0 counts [5, 18]. This change was made to keep the threshold consistent with the VM cut-point for sedentary time [18].

The third method used the algorithms developed by Choi et al. [11, 19]. For vertical axis data, non-wear time is defined as 90-consecutive minutes of 0 cpm, allowing up to a 2-minute interval of non-zero cpm if the interruption is accompanied by 30 consecutive minutes of 0 cpm either up or downstream [11]. For the algorithm using VM data, the same criteria described above were applied [19]. Both algorithms are available in the PhysicalActivity package in R statistical software http://cran.r-project.org/web/packages/PhysicalActivity/index.html [20] and in the ActiLife software.

### Estimation of sedentary time and physical activity

The following summary metrics were estimated for the vertical axis: 1) Minutes the monitor was worn on valid days; 2) Sedentary time (vertical axis cpm <100) [21]; 3) Light activity (vertical axis cpm between 100 and 1951) [12] time; 4) MVPA (vertical axis cpm greater than 1952) [12] time.

The following summary metrics were estimated for the VM: 1) Minutes the monitor was worn on valid days; 2) Sedentary time (VM cpm <200) [18]; 3) Light activity (VM cpm between 200 and 2689) [22] time; 4) MVPA (VM cpm ≥2690) [22] time.

### Data analyses

The data were not normally distributed and are thus presented as medians (interquartile range). The conventional ≥10-hr criteria was applied for determining if wear-time was sufficient to consider the data valid for that day [5]. For each wear-time assessment method, the summary metrics were calculated for all participants with at least one valid day (≥10 h wear). The median number of valid days, number and percent of eligible sample with ≥1 valid day, and ≥4 valid days (conventionally regarded as the minimum needed for validly estimating habitual PA levels [9]) also was calculated. The Wilcoxon signed rank sum test was used to compare the output between vertical and VM axis, within each wear-time equation.

## Results

### Sample size and wear time

The average age of the women was 71.1 (SD = 5.8; range: 62.5, 98.8) years. Additional characteristics of these women have been published elsewhere [7]. For the monitor-logs, the percent of missing data for each of the seven days was examined (Table 1). At day 7, 2.2% of participants were missing dates, 4.3% were missing times, and 23.4% were missing AM/PM for time. Because of the substantial missing data for AM/PM, a series of reasoned assumptions were made to recover as much data as possible (See Additional file 2 for a full description of this process). Even after this imputation, using the detailed-log resulted in only 6834 participants with at least one valid day (Table 2), which was approximately 9% lower than the sample sizes obtained when employing the Troiano (7435, vertical; 7458, VM) and Choi (7485, vertical; 7494, VM) algorithms. Using the detailed-log, of the participants who wore the monitor at least one day, 98% had 4 or more valid days and 93.4% had 6 or more valid days.

**Table 1:** **Description of missing data in participant monitor-logs: Women’s Health Study**

Day	Any missing		
	Date N (%)	Time N (%)	AM or PM N (%)
**One**	2 (0.0)	107 (1.4)	1475 (19.3)
**Two**	51 (0.7)	157 (2.1)	1560 (20.4)
**Three**	66 (0.9)	209 (2.7)	1603 (21.0)
**Four**	79 (1.0)	264 (3.5)	1698 (22.2)
**Five**	102 (1.3)	297 (3.9)	1736 (22.7)
**Six**	120 (1.6)	320 (4.2)	1760 (23.0)
**Seven**	167 (2.2)	329 (4.3)	1788 (23.4)

Note: Date refers to missing month, day or year for a given day; Time refers to missing hour and/or minute for either the time the monitor was put on or the time the monitor was taken off; AM or PM refers to missing AM/PM for either the time the monitor was put on or the time the monitor was taken off.

**Table 2:** **Accelerometer wear time estimates using data from vertical axis only and from vector magnitude assessment, Women's Health Study, 2011-2013**

Assessment method:	Women with ≥1 valid day		Women with ≥4 valid days		Number of valid days		Wear-time on valid days (min)	
	N (%)		N (%)		Median (IQR)		Median (IQR)	
	Vertical	Vector magnitude	Vertical	Vector magnitude	Vertical	Vector magnitude	Vertical	Vector magnitude
Detailed-log	6834 (89.3)	6834 (89.3)	6741 (88.1)	6741 (88.1)	7 (7, 7)	7 (7, 7)	898.0 (850.7, 937.4)	898.0 (850.7, 937.4)
Troiano [5]	7435(97.2)	7458 (97.5)	7202 (94.1)	7312 (95.6)	7 (7, 8)	8 (7, 9)	838.3 (786.8, 886.7)	860.0 (812.5, 904.0)
Choi [11, 19]	7485 (97.8)	7494 (98.0)	7360 (96.2)	7378 (96.4)	9 (8, 9)	9 (8, 10)	875.7 (832.3, 917.0)	887.0 (845.2, 928.2)
Limited-log + Troiano	7383 (96.5)	7391 (96.6)	7110 (96.9)	7188 (94.0)	7, (6, 7)	7 (7, 7)	842.3 (788.7, 890.9)	866.8 (813.3, 912.3)
Limited-log + Choi	7396(96.7)	7396 (96.7)	7247(94.7)	7258 (94.9)	7 (7, 7)	7 (7, 7)	890.4 (841.6, 933.6)	896.4 (848.4, 939.5)

Note: Detailed-log refers to data from participant logs that make use of date and time (hour, minute, Am/PM) that the monitor was put on and off.

Limited-log refers to data from participant logs that make use of date only (no time information used).

Limited-log + algorithm used date of wear from participant logs and time on/off from respective algorithm.

Valid days are defined by convention as those with ≥10 hours wear-time.

Percent values are the N divided by 7650 (eligible sample).

Different wear-time assessment methods yielded different median number of valid days (Table 2). In the study, women were instructed to wear the monitor for 7 days; however, using algorithms resulted in a substantial proportion with greater than 7 days of wear (27.2% Troiano algorithm and 78.7% Choi algorithm). In contrast, 0.2% of women reported wearing the monitor for more than 7 days on their detailed-logs (Table 1). Thus, these results indicated that the algorithms falsely identified days when the monitor was in transit in the mail as days of wear. Therefore, subsequent analyses using the algorithms were restricted to using only the dates provided on the log (without consideration of time on/off from the log; hereafter referred to as the "limited-log"). The algorithms combined with the restricted dates are referred to as "limited-log + Troiano" and "limited-log + Choi". Both algorithms, even when restricted to limited-log dates, yielded larger useable samples than when using the detailed-log (5.1% to 7.6% more women, depending on algorithm and axis) (Table 2).

The median daily wear time estimated from limited-log + Troiano algorithm was lower than those under limited-log + Choi (vertical, 48.1 min lower; VM, 29.6 min lower) (Table 2). Daily wear-time estimates were similar using limited-log + Choi and detailed-logs (Table 2). Within each wear-time assessment method, the sample size with ≥1 or ≥4-valid days and the median days of wear were similar using either vertical axis or VM data, as were median wear-times (difference ranged from 6 min [limited-log + Choi] to 24.6 min [limited-log + Troiano]).

### Sedentary time and physical activity

Sedentary time estimated using limited-log + Troiano (530.1 min, vertical axis) was substantially lower than using detailed-logs (598.7 min, vertical axis) or limited-log + Choi (581.6 min, vertical axis). Estimates of time in light activity and MVPA were similar across all wear-time-methods (Table 3). For sedentary time, the differences in results between axes were much greater than differences across wear-time methods. With detailed-logs, estimates of sedentary time were 80.2 minutes lower using VM data compared to vertical axis data, and 55.5 min and 75.6 min lower using limited-log + Troiano and limited-log + Choi, respectively (Table 3).

**Table 3:** **Physical activity and sedentary behavior estimates using data from vertical axis only and from vector magnitude assessment, Women's Health Study, 2011-2013**

Assessment method:	Sedentary ^a^ (min/d)		Light-intensity ^b^ (min/day)		MVPA ^c^ (min/day)	
	Vertical axis	Vector magnitude	Vertical axis	Vector magnitude	Vertical axis	Vector magnitude
Detailed-log	598.7 (537.3, 656.9)	518.5. (453.4, 585.3)	277.2 (227.6, 330.7)	343.8 (283.9, 404.0)	8.9 (2.7, 21.1)	19.7 (8.0, 37.7)
Limited-log + Troiano [5]	530.1 (480.1, 578.6)	474.6 (417.0, 529.6)	290.4 (242.2, 342.7)	358.7 (300.2, 418.4)	9.6 (3.0, 22.3)	20.7 (8.7, 38.7)
Limited log + Choi [11, 19]	581.6 (521.1, 639.8)	506.0 (439.2, 570.9)	287.9 (238.7, 340.7)	357.4 (297.4, 417.6)	9.4 (3.0, 22.1)	20.6 (8.7, 38.6)

Note: All differences between vertical axis and vector magnitude (VM) were statistically significant P < 0.001. Includes all participants with at least 1 valid day (≥10 h wear-time) for particular assessment method.

^a^Sedentary time is defined as time during which the accelerometer registers vertical cpm <100 [21] and VM cpm <200 [18].

^b^Light-intensity physical activity time is defined as time during which the accelerometer registers vertical cpm between 100 and 1951 [12] and VM cpm between 150 and 2690 [22].

^c^MVPA time is defined as time during which the accelerometer registers vertical cpm > = 1952 [12] and VM cpm > = 2691 [22].

**Figure 2:**
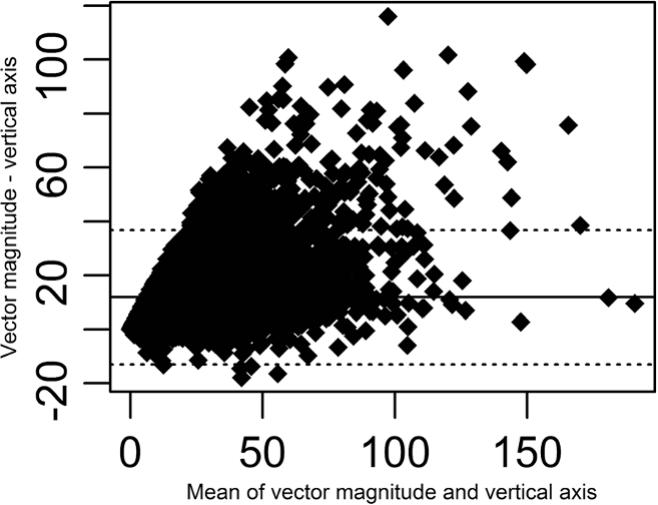
**Bland-Altman plot of MVPA (min/d) for vertical axis and vector magnitude.** Note: Solid line is mean bias and dashed lines are 95% limits of agreement. MVPA is defined as time during which the accelerometer registers vertical cpm > = 1952 [12] and VM cpm > = 2691 [22]. Monitor-wear time was estimated using Limited-log + Choi [10, 19].

For light intensity physical activity, there were significant differences (irrespective of wear-time assessment method) between the vertical axis and VM data that mirrored the differences observed for sedentary time but in the opposite direction (p < 0.001); VM estimates of time in light-intensity activity were ~70 min higher than estimates using vertical axis data (Table 3). Estimates of MVPA time were significantly different between vertical axis and VM data, with medians of approximately 9 min and 20 min, respectively (Table 3). Figure 2 illustrates this graphically for individual participants using limited-log + Choi; the estimates of MVPA were on average 11.9 min higher using VM compared to vertical axis data, with 95% limits of agreement ranging from -13.0 min to 36.8 min. Similar patterns were observed with detailed-logs and with limited log + Troiano.

Age, BMI, smoking status, and PA levels did not influence the results above (See Table, Additional file 3; Differences in estimates of valid minutes by wear-time assessment method and monitor filter across sub-groups).

## Discussion

This study provides data examining the impact of different wear-time assessment algorithms on PA measures obtained using accelerometers in a large sample of over 7,500 women. The results showed that choice of wear-time algorithm impacts: 1) sample size eligible for analysis; 2) estimates of wear-time; and 3) estimates of PA variables. In addition, estimated sedentary time, light-intensity activity and MVPA were substantially different when using vertical axis data alone versus VM data.

Requiring participants to accurately complete detailed-logs so that the data can be used is challenging. To be able to utilize detailed-log data, seven pieces of information are required for each day (valid date, "on" time [hour, minute and am/pm], "off" time [hour, minute and am/pm]). If any of the required variables are missing for a given day, either the data cannot be used for that day, or the data have to be imputed. Participants recorded dates and times much more frequently (missing data <5%) than AM/PM indicators. If AM/PM were not imputed, (see Additional file 2 for assumptions), ~20% of all data would have been missing (Table 1). In total, about 18% of participants who had monitor data were excluded from analyses using detailed-logs.

Additionally, the process of computerizing data from detailed-log is burdensome. In large observational studies, it is infeasible to manually check all instances of incomplete/incongruous data, and tracking monitor delivery dates via mail (to ascertain dates in mail transit) is cost prohibitive. Peeters et al. [10] showed that the participant logs were less accurate than automated algorithms; however, because subjects were handed their monitor directly, investigators did not have to be concerned about "mail-noise". To our knowledge, this is the first study demonstrating that algorithms incorrectly indicated the monitor to be worn when it was actually recording movements in the mailing process. Thus, with mail-based protocols, a combined approach (limited-log + Choi or limited-log + Troiano) is optimal, resulting in useable data from more subjects than using the detailed-log. Future research should examine whether only a record of the first day of wear is required (to further minimize participant burden) and whether new algorithms can correctly identify time spent in the mail.

Across different wear-time methods, estimates of sedentary time differed by over an hour, while differences in MVPA were less than 1 min. Other studies [23, 24] also have reported differences in estimates of sedentary time but minimal effect on estimates of MVPA [25] when wear-time methods are compared. This is to be expected since sedentary time is far longer than time spent in MVPA. In this study, using the Choi algorithm resulted in more wear-time than the Troiano algorithm. The Choi algorithm was empirically derived and validated, and specifically designed to overcome misclassification of wear-time as non-wear time that occurs using the Troiano algorithm, particularly between 11 pm and midnight [11, 23]. While the difference between algorithms was attenuated when VM data were used, estimates still remained lower using the Troiano algorithm. Wear-time estimates using limited-log + Choi closely matched those obtained using detailed-logs; thus the Choi algorithms (either vertical or VM) for estimating wear-time are recommended.

New methods are continuously developed to improve estimates of physical activity and it is important to employ these novel methods in future studies. However, it is also important to empirically evaluate how new methodologies compare to older methods and systematically document the impact on summary estimates. A novel feature of this investigation was quantifying differences in sedentary, light intensity and MVPA time using vertical compared to VM data. This study cannot address which is preferable since there was not a concurrent criterion (or gold standard) measure. However, it is important to note the substantial differences in estimates even though vertical axis and VM linear-regression cut-points were developed using the same treadmill-based protocol [12, 22]. The VM cut-point estimates of MVPA were higher than vertical axis cut-points, likely because activities of daily living require a greater (relative) contribution of motion in the anterior-posterior and medio-lateral axis, compared to locomotion, for which vertical axis motion is the primary contributor [22]. The Freedson et al. equation consistently underestimates MVPA [26], though a criterion measure within a free-living environment is needed to compare the methods directly. In the present sample, there were large differences (~70 min) in sedentary time estimates between vertical axis and VM data. Validation studies have consistently shown that ActiGraph cut-points to define sedentary time are imprecise [27, 28]. If the VM cut-points were used (without comparison using vertical axes data), the data would have shown that participants spent 100 minutes less in sedentary time and double the time in MVPA compared to another analysis on the same population using the vertical axis only data. This example, comparing only VM versus vertical axis data, can be extended to more sophisticated processing methods, including multiple-regression models [29] and machine learning [30, 31].

This study has important strengths. It included a direct comparison of wear-time algorithms and vertical vs. VM axis, which has not been reported previously. Additionally, examination of the efficacy of automated algorithms in a mail-based protocol is novel. Further, the sample size used is substantially larger than previous studies that have compared monitor processing techniques [8, 10, 24, 25]. However, there are also limitations. This study included only older women, which limits generalizability. The study did not employ a gold-standard assessment for time in various PA intensities. However, the intent of the present study was to quantify the impact of different processing decisions in large-scale epidemiologic studies, where it is impossible to directly observe ~7500 participants, rather than validate a particular method. The study compared the two most commonly used algorithms, rather than exhaustively comparing all wear-time algorithms in the literature [5, 11, 23, 32, 33]. Additional data processing decisions were not addressed, including what constitutes valid wear for a day (i.e., number of hours), how many days of wear are needed, and the epoch length [8], which may impact estimates of PA [34–37]. ActiGraph also has a "low-frequency extension" (LFE) filter option, which is recommended for use in older populations. However, the normal filter option was used in the present study since a previously published report from this sample showed that LFE steps/day were implausibly high (8000 steps/day higher than the normal filter [7]; the average US adult takes only 6540 +/- 106 steps/day [38]). Activity monitor technology and protocols are rapidly changing. In the future, monitors that are worn 24-hrs per day for extended periods with a sensor to detect wear-time will likely be widely available; thus, wear-time algorithms may not be needed [39, 40]. However, the results of the current study are directly relevant to several ongoing epidemiologic investigations where data are collected using a standard protocol [7, 24, 41]. Further, the description of physical activity and sedentary time in this large sample of older women can serve as a comparator as new technologies to assess physical activity are introduced to epidemiologic research.

## Conclusions

When a mail-based protocol is used to both send out and receive devices, a participant log is needed to determine dates (but not times) the monitor was worn. Using the automated Choi algorithm to estimate on/off times, combined with use of this limited-log, is feasible and maximizes sample size. Researchers comparing results across studies using vertical axis only or VM data should be aware of large differences in estimates of time in PA and sedentary behavior that can occur. The field of PA epidemiology is moving forward and is increasingly utilizing technology to assess PA more precisely in large-scale studies. However, the lack of consensus on standard processing techniques for accelerometer data is a major challenge [5, 6, 8, 33]. The development of consensus processing procedures is critical for more efficient data processing and facilitating comparisons, as well as pooling data, across studies.

## Electronic supplementary material

Additional file 1: **Activity monitor log.** (PDF 152 KB)

Additional file 2: **Data processing assumptions for monitor-log.** (PDF 81 KB)

Additional file 3: **Differences in estimates of valid minutes by wear-time assessment method and monitor filter across sub-groups.** (PDF 79 KB)

## Abbreviations

PA: Physical activity
MVPA: Moderate-to-vigorous physical activity (greater than or equal to 3 metabolic equivalents)
CPM: Counts per minute
VM: Vector magnitude (square root of the sum of squares of cpm from all 3 axes of accelerometer output).
