# Supplementary material for: Impact of accelerometer data processing decisions on the sample size, wear time and physical activity level of a large cohort study
Source: BMC Public Health. 2014 Nov 24;14:1210. doi: 10.1186/1471-2458-14-1210 (PMC4247661; doi:10.1186/1471-2458-14-1210)
Supplement: Supplementary file 1 — Additional file 1: Activity monitor log. (PDF 152 KB) [file 12889_2014_7313_MOESM1_ESM.pdf]

Study ID: «ID_» **Activity Monitor Log**

«ID_»

Day	Circle the Day of the Week	Date monitor put on	Time out of bed in the morning	Time monitor put on in the morning	Time monitor removed at night	Time into bed for the night	List times during the day the monitor was not worn and state reason for not wearing it (e.g., showering). Any additional comments?
1	M T W TH F SA SU	___/___/___ mm/dd/yy	___ :___ am pm	___ :___ am pm	___ :___ am pm	___ :___ am pm	__________
2	M T W TH F SA SU	___/___/___	___ :___ am pm	___ :___ am pm	___ :___ am pm	___ :___ am pm	__________
3	M T W TH F SA SU	___/___/___	___ :___ am pm	___ :___ am pm	___ :___ am pm	___ :___ am pm	__________
4	M T W TH F SA SU	___/___/___	___ :___ am pm	___ :___ am pm	___ :___ am pm	___ :___ am pm	__________
5	M T W TH F SA SU	___/___/___	___ :___ am pm	___ :___ am pm	___ :___ am pm	___ :___ am pm	__________
6	M T W TH F SA SU	___/___/___	___ :___ am pm	___ :___ am pm	___ :___ am pm	___ :___ am pm	__________
7	M T W TH F SA SU	___/___/___	___ :___ am pm	___ :___ am pm	___ :___ am pm	___ :___ am pm	__________

8127

Thank you for completing this form. Please return it with the activity monitor.

OFFICE USE ONLY ○ 1 ○ 2 ○ 3
