# Supplementary material for: Impact of accelerometer data processing decisions on the sample size, wear time and physical activity level of a large cohort study
Source: BMC Public Health. 2014 Nov 24;14:1210. doi: 10.1186/1471-2458-14-1210 (PMC4247661; doi:10.1186/1471-2458-14-1210)
Supplement: Supplementary file 2 — Additional file 2: Data processing assumptions for monitor-log. (PDF 81 KB) [file 12889_2014_7313_MOESM2_ESM.pdf]

Additional File 2: To prepare the monitor logs, we made the following reasoned assumptions.

If the date was missing and the following criteria were met: 1) there were data for at least one of the time indicators for that day, and 2) there was a previous date recorded by the participant, then the missing date was assumed to be the date following the previous date. (e.g., if day 2 was missing a date and day 1 was 4/16/2013, then the date for day 2 was assumed to be 4/17/2013).

When AM/PM was missing for time the monitor was put on in the morning:

- If the participant indicated AM/PM for the time out of bed, the AM/PM for the time was on monitor was automatically assigned the same value.
- If the participant reported the time on in military time (hour greater than 12), then PM was assigned.
- If the participant did not indicate AM/PM for the time out of bed, the time the monitor was put on was automatically assigned AM.

When AM/PM was missing for time the monitor was taken off in the evening:

- If the participant indicated AM/PM for the time into bed, the AM/PM for the time was on monitor was automatically assigned the same value.
- If the participant reported the time off in military time (hour greater than 12), then PM was assigned.
- If the participant did not indicate AM/PM for the time out of bed, and reported time off between 12 and 4, then AM was assigned.
- If the participant did not indicate AM/PM for the time out of bed, and reported time off between 5 and 11, then PM was assigned.

If the participant indicated she put the monitor on in the evening (e.g., 9:00 PM) and took it off in the morning (e.g., 6:00 AM), we assumed overnight wear and added a day to the “day on” variable to get date off (i.e., date on 4/16/2013 9:00 PM, date off 4/17/2013 6:00 AM).

If wear-time was negative and the time on/off was 12:00, we assumed this reflected differences in recording AM/PM for noon/midnight.

- If time on was exactly 12:00, we assumed PM (noon)
- If time off was exactly 12:00, we assumed AM (midnight)
