# Supplementary material for: Impact of accelerometer data processing decisions on the sample size, wear time and physical activity level of a large cohort study
Source: BMC Public Health. 2014 Nov 24;14:1210. doi: 10.1186/1471-2458-14-1210 (PMC4247661; doi:10.1186/1471-2458-14-1210)
Supplement: Supplementary file 3 — Additional file 3: Differences in estimates of valid minutes by wear-time assessment method and monitor filter across sub-groups. (PDF 79 KB) [file 12889_2014_7313_MOESM3_ESM.pdf]

Additional File 3: Differences in estimates of valid minutes by wear-time assessment method and monitor filter across sub-groups

Subgroups Categorized by:	Detailed-Log	Limited-Log + Troiano		Limited-Log + Choi	
		Vertical	Vector Magnitude	Vertical	Vector Magnitude
**Age, y**					
< 60	903.8 (861.5, 941.9)	865.3 (815.3, 907.2)	886.0 (836.1, 925.5)	903.1 (855.0, 941.7)	906.2 (860.2, 947.1)
60 to <70	902.0 (857.7, 941.6)	853.0 (803.3, 898.9)	875.8 (826.7, 918.5)	895.1 (849.4, 938.1)	901.4 (854.2, 943.4)
≥70	891.9 (844.7, 933.9)	826.4 (776.1, 874.2)	855.1 (802.2, 900.9)	882.4 (833.6, 927.0)	890.6 (843.5, 933.7)
p for trend	<0.01	<0.01	<0.01	<0.01	<0.01
**BMI, kg/m^2**					
<25.0	902.5 (858.5, 941.1)	855.0 (805.8, 900.8)	880.6 (858.5, 921.9)	898.4 (852.2, 939.1)	903.6 (857.7, 944.0)
25 to <30	895.2 (849.1, 935.0)	835.8 (787.6, 881.0)	861.0 (812.2, 903.9)	885.5 (838.9, 928.6)	892.1 (845.6, 935.8)
≥30	888.8 (840.7, 932.8)	813.4 (762.5, 866.2)	839.7 (783.7, 890.7)	873.6 (823.9, 920.9)	882.6 (834.5, 928.8)
p for trend	<0.01	<0.01	<0.01	<0.01	<0.01
**Meeting PA guidelines**					
Yes	889.4 (842.3, 931.5)	834.5 (782.7, 881.5)	858.8 (805.5, 903.0)	882.9 (834.6, 928.2)	890.2 (841.3, 934.9)
No	902.0 (857.3, 941.7)	846.5 (797.4, 892.2)	871.1 (822.6, 913.7)	893.3 (848.5, 935.5)	900.2 (855.0, 942.3)
p for trend	<0.01	<0.01	<0.01	<0.01	<0.01
**Smoking**					
Never	898.6 (854.5, 936.2)	842.4 (793.5, 886.5)	867.8 (819.7, 910.5)	890.8 (846.5, 932.6)	897.6 (852.4, 939.4)
Former	896.1 (849.4, 937.3)	839.8 (790.4, 887.7)	863.7 (812.9, 909.0)	888.5 (840.0, 931.6)	894.7 (846.0, 937.5)
Current	894.6 (843.9, 947.9)	841.9 (779.7, 895.2)	863.8 (806.1, 913.8)	882.4 (833.3, 937.6)	896.2 (843.4, 944.4)
p for trend	ns	ns	ns	ns	ns

Note: Detailed log refers to data from participant logs that make use of date and time (hour, minute, AM/PM) that the monitor was put on and off.
Limited log refers to data from participant logs that make use of date only (no time information used)
Limited-log + algorithm uses date of wear from participant logs and time on/off from respective algorithm. Choi is algorithm from Choi et al [11, 19]. Troiano is algorithm from Troiano et al [5].

Valid days are defined by convention as those with ≥ 10 hours wear-time
Values are median (25th percentile, 75th percentile) and adjusted for age, BMI, and smoking status.
Age, weight, height, PA levels and smoking were self-reported on annual questionnaires. Meeting PA guidelines was defined as at least 150 min/week of moderate intensity, based on 2008 Physical Activity Guidelines [1].
